# Reconsidering the Mechanism of Proton Pump Inhibitor‐Associated Fundic Gland Polyps: Absence of Re‐enlargement during Potassium‐Competitive Acid Blocker Therapy

**DOI:** 10.1002/deo2.70463

**Published:** 2026-11-01

**Authors:** Yoshitsugu Misumi, Tatsuyuki Marumo, Shinichi Ban, Kouichi Nonaka

**Affiliations:** ^1^ Department of Digestive Endoscopy Tokyo Women's Medical University Hospital Tokyo Japan; ^2^ Department of Gastroenterology Ina Hospital Saitama Japan; ^3^ Department of Pathology Dokkyo Medical University Saitama Medical Center Saitama Japan

**Keywords:** esophagogastroduodenoscopy, fundic gland polyps, gastrin, potassium‐competitive acid blockers, proton pump inhibitors

## Abstract

Fundic gland polyps (FGPs) may enlarge during long‐term proton pump inhibitor (PPI) therapy, potentially in association with acid suppression and hypergastrinemia. Potassium‐competitive acid blockers (PCABs) provide potent and sustained acid suppression and may induce comparable or greater elevations in serum gastrin levels than PPIs. We report a case of FGPs in which regression was documented after changes in acid‐suppressive therapy, with no subsequent re‐enlargement during long‐term use of PCABs. A woman in her 60s with gastroesophageal reflux disease developed progressive enlargement of FGPs during 7 years of rabeprazole therapy. Rabeprazole was discontinued because of marked redness of the enlarged FGPs and concern for possible bleeding and was replaced with an H2‐receptor antagonist. After reintroduction of rabeprazole, treatment was switched to a PCAB because of insufficient symptom control. Four months after PCAB initiation, esophagogastroduodenoscopy demonstrated marked regression of the FGPs, with no subsequent re‐enlargement during continued PCAB therapy. Serum gastrin levels were elevated at both measurements during PCAB use (491.9 and 753.7 pg/mL). Histological examination revealed similar fundic gland changes characteristic of FGPs in both a residual polyp and the endoscopically non‐polypoid background mucosa, without dysplasia. Because the exact timing of FGP regression could not be determined, the regression observed 4 months after vonoprazan initiation cannot be solely attributed to vonoprazan therapy and may have occurred, at least in part, during the preceding period of PPI discontinuation. Nevertheless, no subsequent re‐enlargement was observed during long‐term vonoprazan therapy.

Clinical Trial Registration: Clinical trial registration was not applicable because this was a single case report.

## 1 Introduction

Proton pump inhibitors (PPIs) and potassium‐competitive acid blockers (PCABs) are widely used and highly effective oral therapies for gastroesophageal reflux disease. However, adverse effects associated with long‐term acid‐suppressive therapy have been increasingly reported, including enlargement of fundic gland polyps (FGPs). Although most FGPs are benign lesions, enlargement may occasionally result in bleeding and clinical concern [1].

The progression of FGPs is thought to be associated with trophic stimulation of fundic gland cells in response to acid suppression‐induced hypergastrinemia [2]. In addition, PCAB therapy has been reported to induce greater elevations in serum gastrin levels than PPI therapy [3].

Nonetheless, there are limited data on the longitudinal changes in FGPs after switching from PPI to PCAB therapy in patients with PPI‐associated FGP enlargement. Here, we report a case in which FGPs that had progressively enlarged during PPI therapy regressed after changing the acid‐suppressive therapy and remained regressed during long‐term use of PCABs.

## 2 Case Report

A woman in her 60s had been treated with rabeprazole 10 mg/day for gastroesophageal reflux disease since year X. *Helicobacter pylori* infection was excluded based on negative results for serum *H. pylori* IgG antibody and the urea breath test. She had no history of *H. pylori* eradication therapy and showed no endoscopic evidence of gastric mucosal atrophy. At the initial esophagogastroduodenoscopy (EGD) performed in year X, a few FGPs were identified in the gastric body (Figure 1a).

**FIGURE 1:**
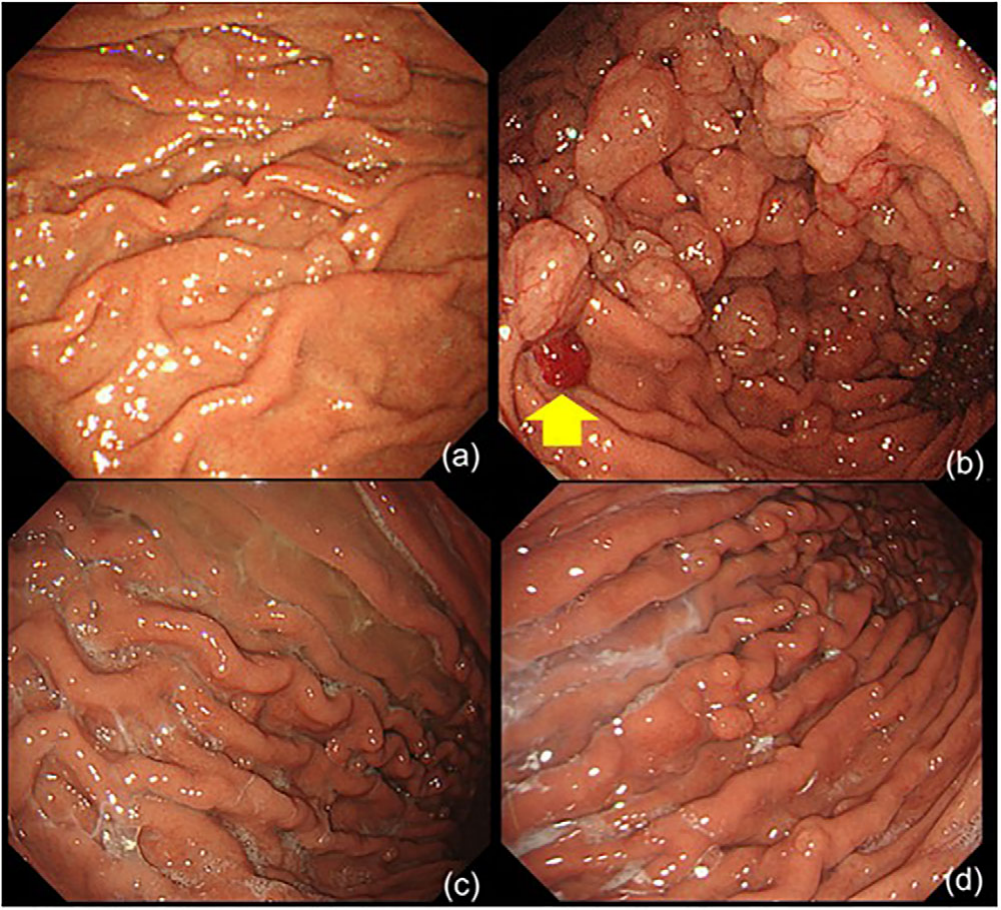
Endoscopic findings showing chronological changes in fundic gland polyps (FGPs) under acid‐suppressive therapy. (a) Initial esophagogastroduodenoscopy revealed a few small FGPs in the gastric body in year X. (b) During long‐term use of proton pump inhibitors, the FGPs progressively enlarged, and some polyps exhibited focal redness, raising concern for bleeding (arrow) in year X+7. (c) Four months after initiation of potassium‐competitive acid blocker (PCAB) therapy, the FGPs showed marked regression. (d) Two years after initiation of PCAB therapy, the FGPs showed no re‐enlargement.

During annual EGD follow‐up under long‐term rabeprazole therapy, the FGPs gradually increased in size and developed edematous changes. In year X+7, EGD revealed marked redness of enlarged FGPs (Figure 1b), raising concern for possible bleeding. Rabeprazole was discontinued and replaced with lafutidine 20 mg/day.

Fourteen months later, reflux symptoms worsened, and rabeprazole 40 mg/day was reintroduced for 9 months without sufficient symptomatic improvement. In year X+9, the treatment was switched from rabeprazole to vonoprazan 20 mg/day, resulting in marked improvement of reflux symptoms. No other medications known to alter gastric acid secretion were used during the clinical course.

EGD performed 4 months after initiation of PCAB therapy demonstrated marked regression of all FGPs (Figure 1c). PCAB therapy was continued, and follow‐up EGD in year X+11 confirmed that the FGPs did not enlarge (Figure 1d).

The overall clinical course, including changes in acid‐suppressive therapy, gastroesophageal reflux disease symptom severity, serum gastrin levels, and the timing of endoscopic examinations, is summarized in Figure 2.

**FIGURE 2:**
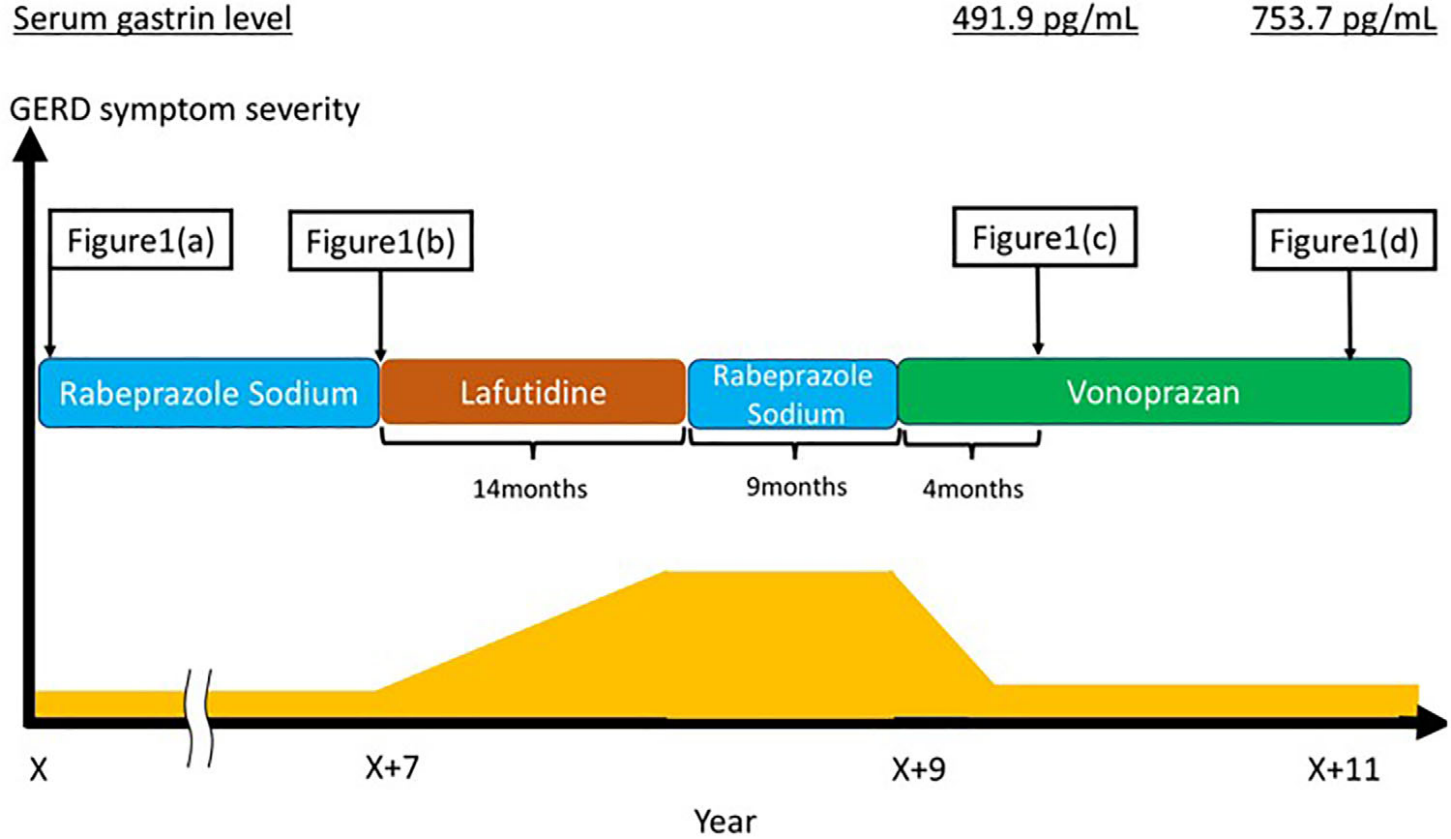
Overall clinical course, including changes in acid‐suppressive therapy, gastroesophageal reflux disease symptom severity, serum gastrin levels, and the timing of endoscopic examinations.

During the EGD performed in year X+11, biopsy specimens were obtained from a residual polyp and the endoscopically non‐polypoid background mucosa (Figures 3a and 4a). Histopathological examination of both specimens revealed cystically dilated fundic glands, shortened gastric pits, and disorganized glandular architecture (Figures 3b,c and 4b,c). No dysplasia was identified.

**FIGURE 3:**
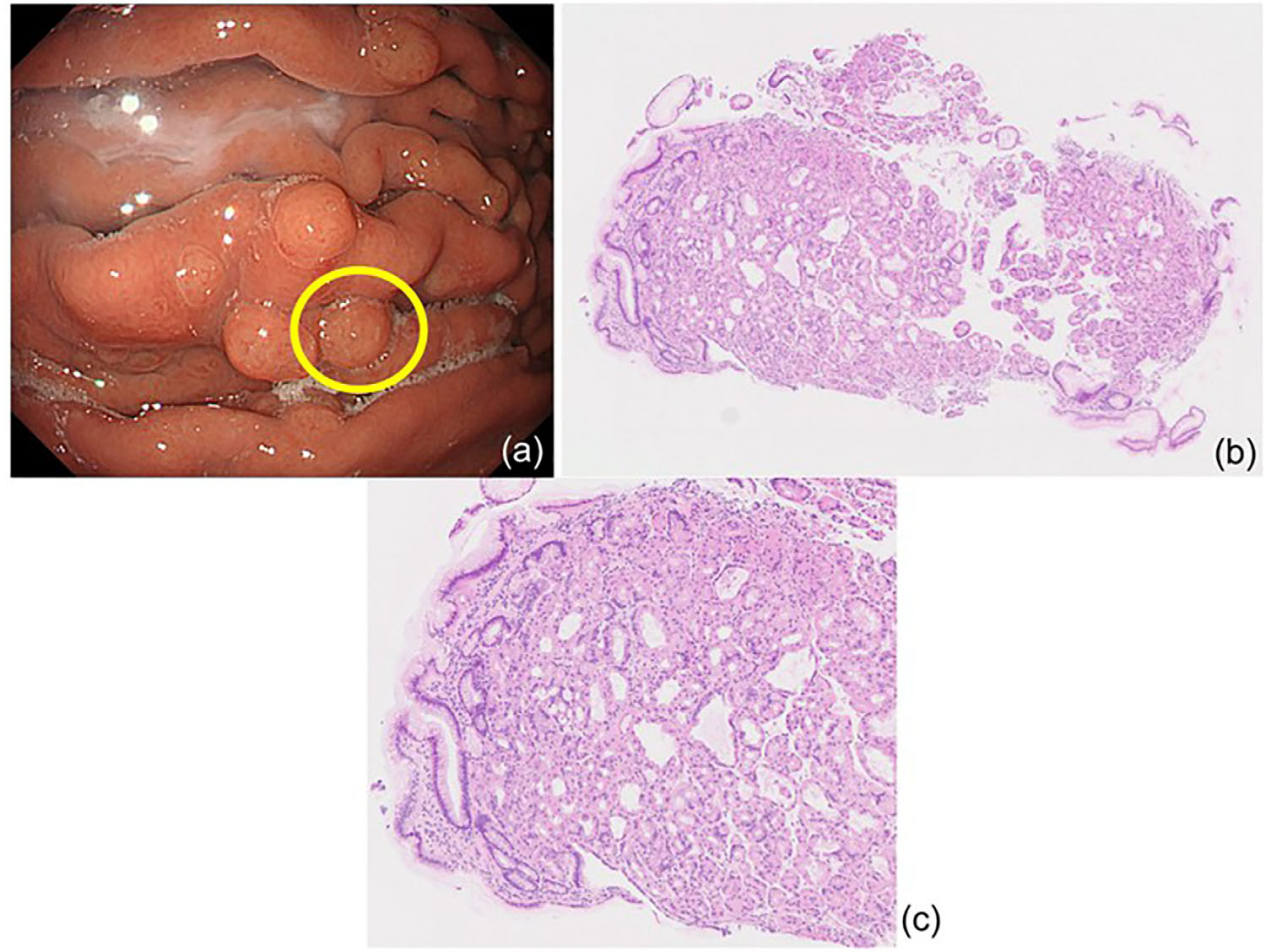
Endoscopic and histopathological findings of a residual fundic gland polyp in year X+11. (a) The circle indicates a representative residual polyp selected for biopsy. (b) Low‐power histopathological image showing the overall glandular architecture. (c) Higher‐power histopathological image demonstrating cystically dilated fundic glands, shortened gastric pits, and disorganized glandular architecture, findings consistent with a fundic gland polyp. No dysplasia is present.

**FIGURE 4:**
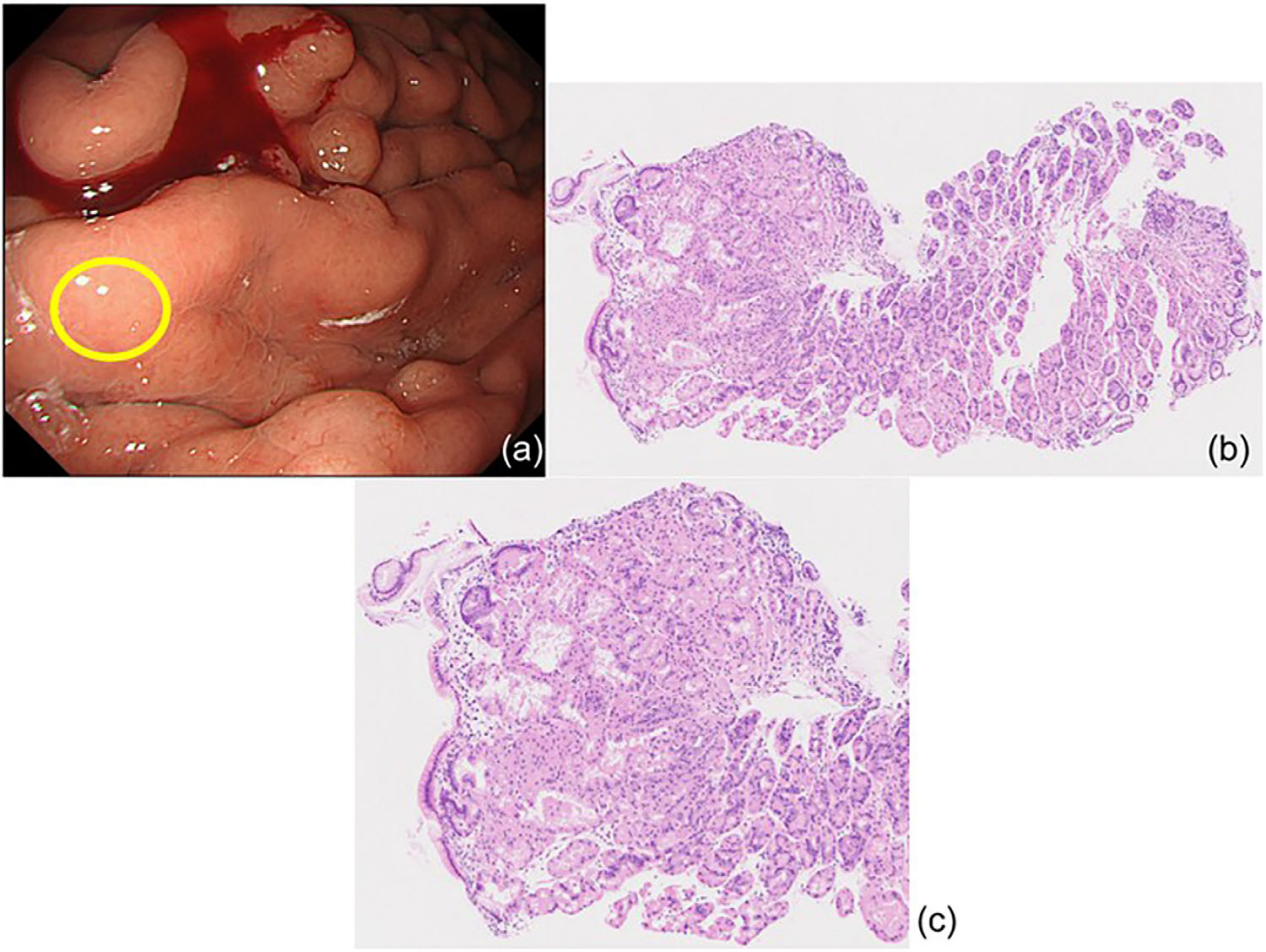
Endoscopic and histopathological findings of the non‐polypoid mucosa in year X+11. (a) Non‐polypoid mucosa selected for biopsy is indicated by the circle. (b) Low‐power histopathological image showing the overall glandular architecture. (c) Higher‐power histopathological image demonstrating cystically dilated fundic glands, shortened gastric pits, and disorganized glandular architecture, similar to the findings in the residual fundic gland polyp (Figure 3c). No evidence of dysplasia is identified.

Serum gastrin levels were 491.9 pg/mL 4 months after PCAB initiation and 753.7 pg/mL in year X+11. The patient remains under regular annual endoscopic surveillance while continuing PCAB treatment.

## 3 Discussion

In the present case, FGPs that had progressively enlarged during long‐term use of PPIs demonstrated marked regression after changing the acid‐suppressive therapy and did not re‐enlarge for more than 1 year during long‐term use of PCABs. This clinical course suggests that the relationship among FGP progression, potent acid suppression, and hypergastrinemia may not be uniform. Because EGD was not performed during lafutidine therapy or after rabeprazole reintroduction, the exact timing of FGP regression cannot be definitively determined. Therefore, the regression documented 4 months after vonoprazan initiation cannot be attributed to vonoprazan therapy and may have occurred, at least in part, during the preceding period of PPI discontinuation. Previous reports have shown that PPI‐associated FGPs may regress after PPI discontinuation [4]. The noteworthy finding in the present case is thus the absence of subsequent re‐enlargement during continued vonoprazan therapy. Previous studies have reported an increased prevalence and number of FGPs during long‐term vonoprazan therapy, supporting an association between potent acid suppression and FGP development [5]. Conversely, regression of FGPs after de‐escalation of acid‐suppressive therapy has also been reported [6]. In contrast to previous reports showing an increase in FGPs during long‐term vonoprazan therapy, the FGPs in the present case remained regressed during continued vonoprazan therapy. Although the duration of vonoprazan therapy in our patient was shorter than that of the preceding PPI therapy, this contrasting clinical course suggests that the longitudinal behavior of FGPs during potent acid suppression may vary among patients. Given that PCABs provide potent and sustained acid suppression and are associated with comparable or greater elevations in serum gastrin levels than PPIs, re‐enlargement of FGPs might have been expected. Serum gastrin levels were elevated at both measured time points during PCAB therapy: 491.9 pg/mL at 4 months, when marked regression of FGPs was documented, and 753.7 pg/mL at subsequent follow‐up, when no re‐enlargement was observed. Although these measurements do not allow assessment of longitudinal gastrin dynamics, marked FGP regression was observed when the serum gastrin level was 491.9 pg/mL, and no re‐enlargement was observed at subsequent follow‐up when the level was 753.7 pg/mL. Although these observations do not clarify the role of hypergastrinemia in FGP pathogenesis, they suggest that the relationship between serum gastrin levels and the macroscopic behavior of FGPs may not be straightforward. Moreover, similar histological changes were observed in both a residual polyp and non‐polypoid mucosa, suggesting that polyp formation may depend on factors beyond these microscopic alterations.

This report has several limitations. First, serum gastrin levels were assessed at only two time points during PCAB therapy and were not measured before PCAB initiation, precluding comparison of serum gastrin levels between PPI and PCAB therapy and limiting assessment of longitudinal gastrin dynamics. Second, histological examination was not performed before PPI discontinuation, precluding comparison with the findings obtained during subsequent PCAB use. Third, the duration of PCAB therapy was considerably shorter than the duration of PPI therapy; therefore, the possibility that prolonged exposure to potent acid suppression and hypergastrinemia might eventually lead to re‐enlargement of FGPs cannot be excluded. Fourth, causal relationships cannot be established from a single case.

Despite these limitations, the longitudinal endoscopic course over approximately a decade, characterized by the absence of re‐enlargement of FGPs during long‐term use of PCABs despite elevated serum gastrin levels and the presence of similar histological changes in non‐polypoid mucosa, suggests that the relationship among these factors and the macroscopic behavior of FGPs may be complex.

In conclusion, this case suggests that the relationship between microscopic fundic gland abnormalities and macroscopic FGP formation or enlargement may be complex. Additional studies with longer‐term follow‐up are warranted to clarify the factors associated with the longitudinal evolution of FGPs.

## Author Contributions

Yoshitsugu Misumi prepared and submitted the manuscript. Tatsuyuki Marumo performed endoscopic follow‐up. Shinichi Ban performed histopathological analyses. Kouichi Nonaka revised the manuscript.

## Funding

The authors have nothing to report. No funding was received for this study.

## Ethics Statement

This case report was conducted in accordance with the Declaration of Helsinki.

## Consent

Informed consent for publication was obtained from the patient.

## Conflicts of Interest

The authors declare no conflicts of interest.
